# Frequency and clinical significance of short cervix in patients with preterm premature rupture of membranes

**DOI:** 10.1371/journal.pone.0174657

**Published:** 2017-03-30

**Authors:** Seung Mi Lee, Kyo Hoon Park, Eun Young Jung, Ji Ae Jang, Ha-Na Yoo

**Affiliations:** 1 Department of Obstetrics and Gynecology, Seoul National University College of Medicine, Seoul, Korea; 2 Department of Obstetrics and Gynecology, Seoul National University Hospital, Seoul, Korea; 3 Department of Obstetrics and Gynecology, Seoul National University Bundang Hospital, Seongnam, Korea; University of Illinois at Urbana-Champaign, UNITED STATES

## Abstract

**Objective:**

Cervical length measurement has been uggested as a useful tool for predicting intra-amniotic infection/inflammation in preterm labor, but little information is available in the setting of preterm premature rupture of membranes (pPROM). We aimed to determine whether a short cervical length is independently associated with an increased risk of intra-amniotic infection or inflammation and impending preterm delivery in women with pPROM.

**Methods:**

This was a retrospective cohort study involving 171 consecutive singleton pregnant women with pPROM (21+0–33+6 weeks’ gestation), who underwent amniocentesis. Amniotic fluid (AF) was cultured, and assayed for interleukin (IL)-6 and IL-8. Cervical length was measured at the time of amniocentesis by transvaginal ultrasonography with an aseptic technique. Short cervical length was defined as a cervical length of ≤15 mm. Intra-amniotic infection was defined as a positive AF culture for microorganisms and intra-amniotic inflammation was defined as elevated AF concentrations of IL-6 or IL-8 (IL-6 ≥1.5 ng/mL and/or IL-8 ≥1.3 ng/mL).

**Results:**

Fifty (29.2%) women had a sonographic cervical length of ≤15mm. On univariate analysis, short cervical length was associated with an increased risk for intra-amniotic infection and/or inflammation; no other parameters studied showed a significant association. Multivariable analyses indicated that short cervical length was significantly associated with a higher risk of impending preterm delivery (within 2 days of measurement, within 7 days of measurement, and before 34 weeks), and remained significant after adjustment for potential confounders.

**Conclusion:**

In women with pPROM, short cervical length is associated with an increased risk for intra-amniotic infection/inflammation and associated with impending preterm delivery, independent of the presence of intra-amniotic infection/inflammation.

## Introduction

Preterm premature rupture of membranes (pPROM) is a precursor in approximately one-third of preterm deliveries, and is associated with significant perinatal mortality and morbidity [1, 2]. Intra-amniotic infection/inflammation is present in 30–60% of women with pPROM, and is associated with an increased risk of impending preterm delivery and adverse neonatal outcomes [3–6].

Cervical length at mid-pregnancy is a useful tool to identify women at high-risk for preterm delivery [7–9]. Several studies have suggested that a short cervix can also be used as a marker for impending preterm delivery in women with preterm labor and intact membranes [10]. In addition, cervical length has been suggested as a useful tool for predicting intra-amniotic infection/inflammation in preterm labor [11, 12].

Although previous studies demonstrated that measurement of cervical length in patients with pPROM does not increase the risk of infection [13, 14], cervical length evaluation is not universally performed in patients with pPROM, and the significance of a short cervix in pPROM is not as well understood as it is in asymptomatic women or in preterm labor. Studies have reported that a short cervix may be also associated with an increased risk of impending delivery in pPROM [14–17]. However, to date, there is a paucity of information regarding the significance of a short cervix in pPROM in terms of intra-amniotic infection/inflammation. In addition, it is also unclear if the increased risk of impending preterm delivery in pPROM associated with a short cervix is related to the presence of intra-amniotic infection/inflammation, as intra-amniotic infection/inflammation also carries a higher risk of preterm delivery. These issues are important because early diagnosis of such conditions in the initial evaluation of pPROM patients is possible by measuring cervical length with a noninvasive and rapid technique.

The purpose of the current study was to determine whether a short cervical length is independently associated with increased risks of intra-amniotic infection or inflammation and impending preterm delivery in women with pPROM.

## Materials and methods

### Study design

This was a retrospective cohort study. The study population consisted of pregnant women who were consecutively admitted with the diagnosis of pPROM (21+0–33+6 weeks of gestation) between June 2004 and April 2015 at Seoul National University Bundang Hospital. Inclusion criteria were: 1) singleton pregnancy; 2) amniocentesis performed to determine the microbial and inflammatory status of amniotic fluid (AF) or fetal lung maturation; and 3) cervical length measured at the time of amniocentesis. Women with prophylactic cerclage early in the pregnancy, active labor (defined by the presence of cervical dilatation >3 cm by sterile speculum examination), multiple-gestation pregnancy, or major fetal congenital anomalies (defined as structural defects of the body and/or organs that impair viability and require intervention) were excluded. At the time of admission, transabdominal amniocentesis and cervical length measurements were offered to women who were admitted with pPROM at our institution during the study period. pPROM was diagnosed based on sterile speculum examination and a combination of AF pooling, nitrazine, and ferning test results. Intra-amniotic infection and/or inflammation was used as the primary outcome measure because maternal and neonatal outcomes of patients with intra-amniotic inflammation, but a negative AF culture, is similar to that of pateints with a positive AF culture in the context of PPROM [6, 18]. Secondary outcomes included preterm delivery within 2 days of measurement, 7 days of measurements, and before 34 weeks. The Institutional Review Board of Seoul National University Bundang Hospital approved the study (project number B-1105/128-102), and patients provided written consent for collection and use of AF samples for research purposes.

### Amniotic fluid

After informed consent, transabdominal amniocentesis was performed under ultrasound guidance. The retrieved AF was sent to the laboratory for culture of aerobic/anaerobic bacteria and genital mycoplasmas (*Ureaplasma urealyticum* and *Mycoplasma hominis*), and for analysis of white blood cell (WBC) counts according to methods previously described in detail [19]. The remaining AF was aliquoted and stored at -70°C after centrifugation until assayed. Interleukin (IL)-6 and IL-8 levels were measured in stored AF using the enzyme-linked immunosorbent assay human DuoSet Kit (R&D System, Minneapolis, MN, USA). The ranges of the IL-6 and IL-8 standard curves were 7.8–600 and 31.2–2000 pg/ml, respectively. All samples were assayed in duplicate. The intra- and inter-assay coefficients of variation were <10% each. Intra-amniotic infection was defined as a positive AF culture for microorganisms; intra-amniotic inflammation was defined as elevated AF concentrations of IL-6 or IL-8 (IL-6 ≥1.5 ng/mL and/or IL-8 ≥1.3 ng/mL) [11]. Intra-amniotic infection/inflammation was defined as a positive AF culture and/or the presence of intra-amniotic inflammation.

### Measurements of cervical length, AF volume, and C-reactive protein

At the time of amniocentesis, transvaginal ultrasonography to measure cervical length was performed by the maternal-fetal medicine fellows or high grade resident physicians, who had been appropriately trained in this measurement. Either an Accuvix XQ (Medison Co. Ltd., Seoul, Korea) or an Envisor (Philips Medical System, Eindhoven, The Netherlands) ultrasound machine was used with a 6.0-MHz transducer covered by a sterile condom. The patient was asked to empty her bladder and placed in the dorsal lithotomy position for measurement. The transvaginal probe was inserted into the vagina using aseptic technique and minimal pressure was adjusted after obtaining an image of the anterior and posterior cervical lip. The length between the internal and external cervical os was measured at least three times as the furthest straight-line distance, and the shortest length was designated as the cervical length. A short cervix was defined as a cervical length ≤15 mm. Amniotic fluid index (AFI), which was calculated according to the technique described by Phelan et al. [20], was measured near the time of amniocentesis by ultrasound (among all 171 participants, 166 women (97.0%) had an ultrasound performed within 1 day of amniocentesis, 3 (1.8%) within 2 days, and 2 (1.2%) within 4 days). Oligohydramnios was defined as an AFI <5.0 cm. C-reactive protein (CRP) in maternal blood was usually measured within 2–3 hours of amniocentesis.

### Management of pPROM and definitions of various factors

Prophylactic antibiotics were given, but the type of antibiotics was left to the discretion of the attending obstetrician. Ampicillin was the main antibiotic used; however, in many cases erythromycin or azithromycin was administered additionally. Tocolytic therapy (magnesium sulfate, ritodrine or atosiban) and corticosteroids were administered at the discretion of the attending obstetrician. Medications such as antibiotics, corticosteroids and tocolytics were started after amniocentesis. Digital examinations were prohibited until the onset of labor (defined by the presence of regular and painful uterine contractions). Histologic diagnoses of chorioamnionitis, funisitis, and clinical chorioamnionitis were made according to definitions previously described in detail [18, 21]. Endometritis was defined as a temperature of ≥38°C on two separate occasions at least 6 hours apart postpartum (excluding the first 24 hours postpartum) in association with uterine tenderness and no other source of infection. Significant neonatal morbidity was defined as the presence of one or more of the following conditions: early-onset sepsis, respiratory distress syndrome (RDS), bronchopulmonary dysplasia (BPD), intraventricular hemorrhage (IVH, ≥ grade 2), periventricular leukomalacia (PVL), or necrotizing enterocolitis (NEC). These conditions were diagnosed according to the definitions previously described in detail [18].

### Statistical methods

Proportions were compared using the χ^2^-test or Fisher’s exact test and comparisons of continuous variables were performed with the Student’s *t*-test or Mann-Whitney U test, as appropriate. Continuous data were assessed for normality using the Shapiro-Wilk test and are expressed as mean and standard deviation (for normally distributed variables) or median and interquartile range (for non-normally distributed variables). A Kaplan-Meier analysis was used to assess the measurement-to-delivery interval and log-rank tests were used to compare groups with respect to the interval to delivery. Univariate Cox regression was used to identify variables with potential prognostic significance. Thereafter, multivariate Cox regression analysis was performed, including gestational age at measurement, parity, intra-amniotic infection/inflammation, and oligohydramnios as covariates with P < 0.05 in univariate Cox regression. Women delivered preterm for maternal or fetal indications were treated as censored observations, with a censoring time equal to the measurement-to-delivery interval. Multiple logistic regression analysis was used to examine the independent predictability of statistically significant univariate variables on the latency from measurement to delivery. All reported P-values are two-sided, with a significance level of 0.05. All statistical analyses were conducted using SPSS for Windows version 21.0 (IBM SPSS Statistics, Chicago, IL, USA) and MedCalc Statistical Software version 13.3.1 (MedCalc Software bvba, Ostend, Belgium).

## Results

During the study period, 180 women met eligibility inclusion criteria; nine were excluded from analysis for the following reasons. Three had a history of cerclage during the index pregnancy. AF samples for cytokine determination were unavailable for 5 women (1 AF-positive and 4 AF-negative cultures). One was lost to follow up. Thus, a total of 171 women with pPROM were included in the final analysis.

**Fig 1:**
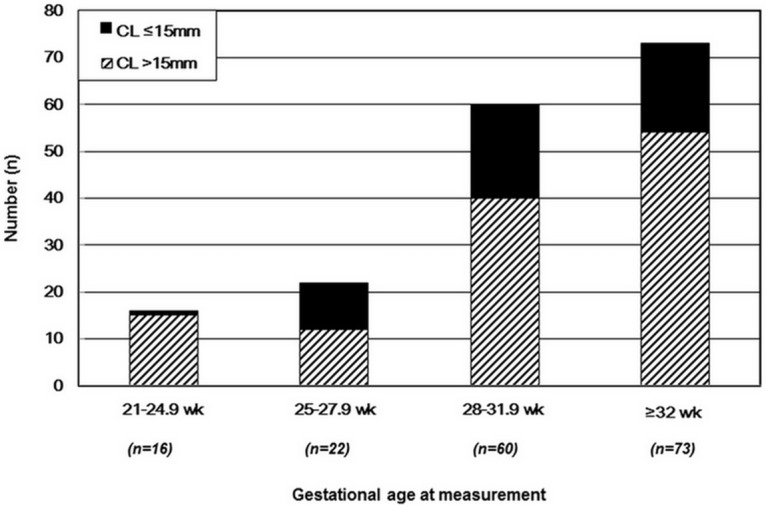
Frequency of a short cervix and normal cervical length stratified by gestational age at measurement (P = 0.052 for χ^2^ test across 4 groups). Closed columns represent a short cervix. Hash-marked columns represent normal cervical length.

The mean (SD) cervical length was 23.9 (12.6) mm and the cervical length was ≤15mm in 50 (29.2%) women. Fig 1 shows numbers of patients with a short cervix and those with normal cervical length stratified by gestational age. The prevalence of positive AF culture and intra-amniotic inflammation were 36.8% (63/171) and 54.4% (93/171), respectively.

**Fig 2:**
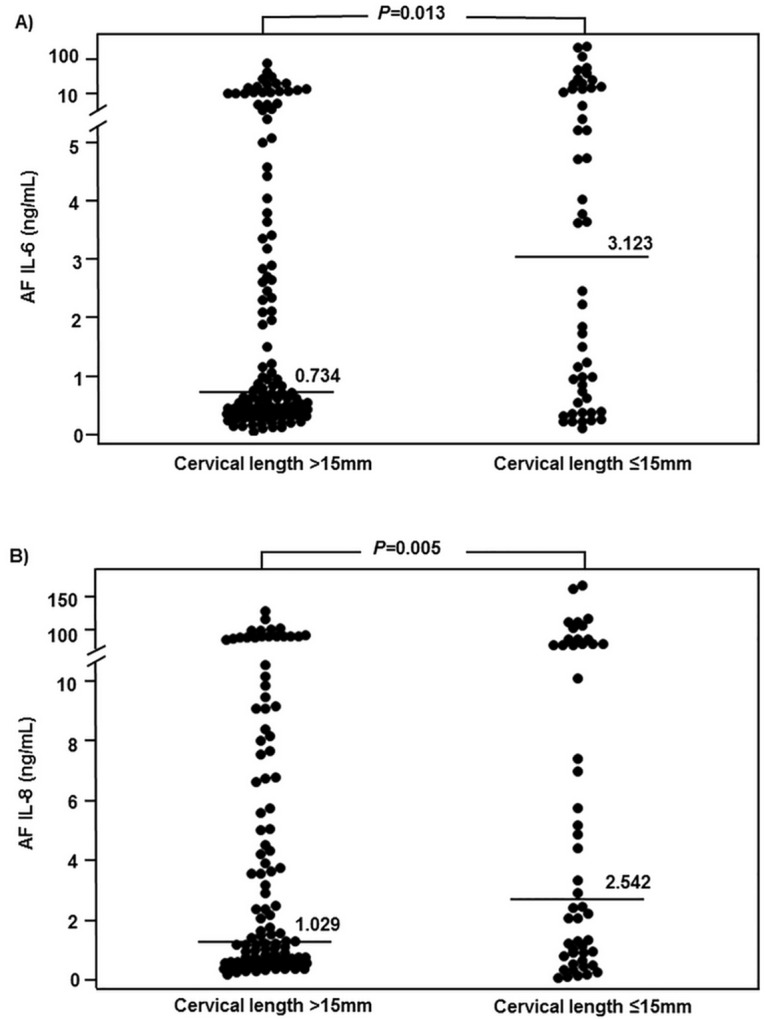
Amniotic Fluid (AF) Interleukin-6 (IL-6) and AF IL-8 concentrations of the study population according to the presence or absence of a short cervix (AF IL-6: Median 3.123 ng/ml, interquartile range 0.569 to 16.181 ng/ml vs. median 0.734 ng/ml, interquartile range 0.378 to 4.096 ng/ml, P = 0.013; AF IL-8: Median 2.542 ng/ml, interquartile range 0.935 to 14.407 ng/ml vs. median 1.029 ng/ml, interquartile range 0.389 to 6.509 ng/ml, P = 0.005; respectively).

Table 1 compares demographic and clinical characteristics of the study population according to the presence or absence of a short cervix. There were no differences in maternal age, rate of nulliparity, gestational age at measurement, CRP levels, AF WBC count, rates of positive AF cultures, oligohydramnios, and steroid, tocolytic or antibiotic treatment. However, women with a short cervix had a significantly higher frequency of intra-amniotic infection/inflammation than those with normal cervical length. Similarly, the median concentrations of AF IL-6 and IL-8 were significantly higher in women with a short cervix than in those with normal cervical length (Fig 2).

**Table 1:** Clinical characteristics of the study population according to the presence or absence of a short cervix.

	Cervical length >15mm (n = 121)	Cervical length ≤15mm (n = 50)	P
Maternal age (years)	32.2 ± 4.2	31.0 ± 3.9	0.110
Nulliparity	59 (48.8%)	24 (48.0%)	1.000
Gestational age at measurement (weeks)	31.4 (28.8–33.0)	31.5 (28.1–32.6)	0.742
<29+0 weeks	30 (24.8%)	16 (32.0%)	0.334
≥29+0 weeks	91 (75.2%)	34 (68.0%)	
Cervical length (mm)	29.9 (23.0–37.0)	10.0 (5.6–12.6)	<0.001
Cervical dilatation at measurement^a^ (cm)	0.5 ± 0.6 (n = 112)	1.0 ± 0.8 (n = 46)	<0.001
Positive AF culture	39/120 (32.5%)	24/50 (48.0%)	0.081
AF IL-6 (ng/mL)	0.734 (0.374–4.160)	3.123 (0.589–16.181)	0.013
AF IL-8 (ng/mL)	1.029 (0.389–6.509)	2.542 (0.935–14.407)	0.005
AF WBC	10 (3–120)	9 (2–965)	0.537
Intra-amniotic inflammation^b^	59/120 (49.2%)	34/50 (68.0%)	0.025
Intra-amniotic infection and/or inflammation	66 (54.5%)	36 (72%)	0.034
Maternal blood CRP	0.34 (0.11–0.69)	0.36 (0.07–1.15)	0.526
Oligohydramnios^c^	31 (25.6%)	9 (18.0%)	0.326
Antibiotics	115 (95.0%)	48 (96.0%)	1.000
Tocolytics	65 (53.7%)	26 (52.0%)	0.838
Antenatal corticosteroids	103 (85.1%)	46 (92.0%)	0.316

AF, amniotic fluid; IL, interleukin; WBC, white blood cell; CRP, C-reactive protein. Values are given as the mean ± standard deviation, median (interquartile range) or n (%).

^a^Thirteen cases were excluded from the analysis because of unavailable data on cervical dilatation.

^b^Intra-amniotic inflammation was defined as elevated AF levels of IL-6 (≥1.5 ng/mL) and/or IL-8 (≥1.3 ng/mL).

^c^Oliohydramnios was defined as an amniotic fluid index <5 cm.

Table 2 shows pregnancy and neonatal outcomes according to the presence or absence of a short cervix. Women with a short cervix delivered significantly earlier and had higher risks of preterm delivery (within 2 days of measurement, within 7 days of measurement, and before 34 weeks) and higher rates of histologic chorioamnionitis than those with normal cervical length. However, the rates of low Apgar scores, admission to neonatal intensive care unit (NICU), and adverse neonatal outcome (neonatal death or significant neonatal morbidity) were similar between the two groups.

**Table 2:** Pregnancy and neonatal outcomes according to the presence or absence of a short cervix.

	Cervical length >15mm (n = 121)	Cervical length ≤15mm (n = 50)	P
Gestational age at delivery (weeks)^a^	33.6 (31.1–34.1) (n = 107)	32.1 (30.2–33.4) (n = 48)	0.001
Measurement-to-delivery interval^a^			
≤48 hours	18/107 (16.8%)	22/48 (45.8%)	<0.001
≤7 days	47/107 (43.9%)	36/48 (75.0%)	<0.001
Preterm delivery <34 weeks^a^	66/107 (61.7%)	43/48 (89.6%)	<0.001
Clinical chorioamnionitis	10/121 (8.3%)	6/50 (12.0%)	0.564
Histologic chorioamnionitis^b^	43/103 (41.7%)	31/48 (64.6%)	0.014
Funisitis^b^	22/103 (21.4%)	15/48 (31.3%)	0.224
Endometritis^c^	1/106 (0.9%)	0/47 (0%)	1.000
Birth weight (g)^c^	2035 (1626–2243) (n = 106)	1770 (1460–2050) (n = 47)	0.023
1-min Apgar score <7^d^	48/104 (46.2%)	29/48 (60.4%)	0.118
5-min Apgar score <7^d^	12/104 (11.5%)	8/48 (16.7%)	0.441
Admission to NICU^d^	94/104 (90.4%)	47/48 (97.9%)	0.175
Neonatal death or significant morbidity^e^	28/104 (26.9%)	17/48 (35.4%)	0.286

PTB, preterm birth; NICU, neonatal intensive care unit. Values are given as the mean ± standard deviation, median (interquartile range) or n/N (%).

^a^ Sixteen women who were lost to follow-up were excluded from this analysis.

^b^Data for histologic evaluation of the placenta were available in 151 (88%) of 171 women because in 18 cases, the delivery took place at another institution and in 2 cases, histologic evaluation of the placenta was not performed because of our institutional policy that only the placentas in cases of preterm delivery are to be sent for histopathologic examination.

^c^Eighteen cases were excluded for the analysis because the delivery took place at another institution and birth weights were unknown.

^d^Nineteen cases were excluded for the analysis because in 17 cases, delivery took place at another institution and 2 newborns were not actively resuscitated at birth due to extremely low gestational age (< 23.0 weeks).

^e^Nineteen infants were excluded from the analysis because we did not have accurate data about their morbidities as the delivery took place at another institution or the infants have been transferred to another institution after admission of NICU.

**Fig 3:**
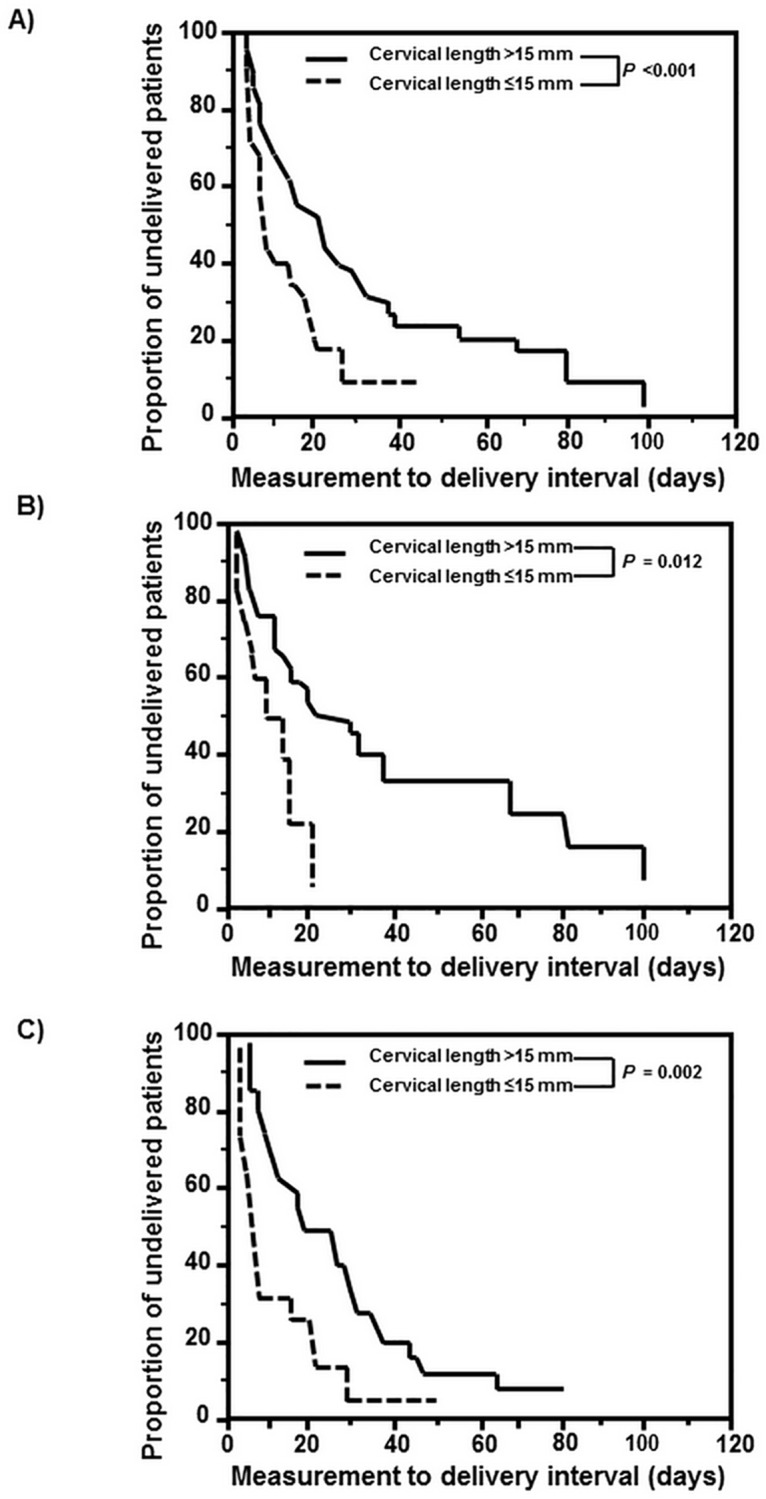
Kaplan-Meier estimates of measurement-to-delivery interval according to the results of cervical length assessed by ultrasound (using 15 mm as cut-off) in (A) total study population (median, 4.08 days [95% CI, 2.59–5.58] vs. 19.29 days [95% CI, 11.24–27.35]; P <0.001), (B) women without intra-amniotic infection/inflammation (median, 6.83 days [95% CI, 0.00–15.21] vs. 18.54 days [95% CI, 1.96–35.13]; P = 0.012), and (C) women with intra-amniotic infection/inflammation (median, 3.25 days [95% CI, 1.78–4.72] vs. 19.79 days [95% CI, 9.04–30.55]; P = 0.002).

Fig 3 displays the measurement-to-delivery interval according to the presence or absence of a short cervix. Women with a short cervix had a significantly shorter measurement-to-delivery interval than those with normal cervical length (Fig 3A). Multivariate analysis with Cox proportional hazards modeling indicated that this result remained significant after adjustment for parity, gestational age at measurement, the presence or absence of intra-amniotic infection/inflammation, and oligohydramnios (hazard ratio: 2.71; 95% CI, 1.74–4.23, P < 0.001). When subgroup analysis was done according to the presence or absence of intra-amniotic infection/inflammation, the relationship between a short cervix and the shorter measurement-to-delivery interval also remained significant in both women with and without intra-amniotic infection/inflammation (Fig 3B and 3C).

As shown in Table 3, short cervical length was a significant and independent predictor of preterm delivery within 2 days of measurement, 7 days of measurement, and before 34 weeks even after adjustment for confounders (parity, gestational age at measurement, intra-amniotic infection/inflammation, and oligohydramnios).

**Table 3:** Relationship of various independent variables with the risk of preterm birth analyzed by multiple logistic regression analysis.

Variables	Odds ratio	95% Confidence interval	P value
**1) Delivery within 2 days of measurement**			
Short cervical length (≤15mm)	4.813	2.044–11.333	<0.001
Nulliparity	0.735	0.321–1.684	0.467
Gestational age at measurement (weeks)	1.399	1.161–1.684	<0.001
Intra-amniotic infection/inflammation^a^	3.004	1.206–7.482	0.018
Oligohydramnios	2.419	0.914–6.402	0.075
**2) Delivery within 7 days of measurement**			
Short cervical length (≤15mm)	4.897	2.061–11.634	<0.001
Nulliparity	0.378	0.175–0.815	0.013
Gestational age at measurement (weeks)	1.426	1.223–1.661	<0.001
Intra-amniotic infection/inflammation^a^	2.227	0.969–5.118	0.059
Oligohydramnios	3.904	1.458–10.454	0.007
**3) Delivery before 34 weeks of gestation**			
Short cervical length (≤15 mm)	5.650	1.924–16.603	0.002
Nulliparity	0.472	0.211–1.055	0.067
Gestational age at measurement (weeks)	0.842	0.716–0.990	0.037
Intra-amniotic infection/inflammation^a^	3.546	1.550–8.115	0.003
Oligohydramnios^b^	1.448	0.546–3.839	0.456

^a^Intra-amniotic inflammation was defined as elevated AF levels of IL-6 (≥1.5 ng/mL) and/or IL-8 (≥1.3 ng/mL).

^b^Oliohydramnios was defined as an amniotic fluid index <5 cm.

Table 4 shows diagnostic values of various cervical lengths, from 10 to 25 mm, in the prediction of intra-amniotic infection/inflammation and preterm birth. As the cut-off point for the cervical length increases, the sensitivity for predicting intra-amniotic infection/inflammation and preterm birth increases; however, the specificity declines.

**Table 4:** Comparison of different cut-off values for cervical length in predicting intra-amniotic infection/inflammation and preterm birth.

Outcome	Cervical length cut-off			
	≤25 mm	≤20 mm	≤15 mm	≤10 mm
**Intra-amniotic infection/inflammation**^a^				
Prevalence % (n)	59.6% (102/171)			
Sensitivity	62.7%	46.1%	35.3%	15.7%
Specificity	58.0%	68.1%	79.7%	88.4%
Positive predictive value	68.8%	68.1%	72.0%	66.7%
Negative predictive value	51.3%	46.1%	45.5%	41.5%
**Delivery within 2 days**^b^				
Prevalence % (n)	25.8% (40/155)			
Sensitivity	82.5%	72.5%	55.0%	27.5%
Specificity	53.9%	68.7%	77.4%	89.6%
Positive predictive value	38.4%	44.6%	45.8%	47.8%
Negative predictive value	89.9%	87.8%	83.2%	78.0%
**Delivery within 7 days**^b^				
Prevalence % (n)	53.5% (83/155)			
Sensitivity	74.7%	60.2%	43.4%	19.3%
Specificity	66.7%	79.2%	83.3%	90.3%
Positive predictive value	72.1%	76.9%	75.0%	69.6%
Negative predictive value	69.6%	63.3%	56.1%	49.2%
**Delivery before 34 weeks**^b^				
Prevalence % (n)	70.3% (109/155)			
Sensitivity	67.0%	52.3%	39.4%	19.3%
Specificity	71.7%	82.6%	89.1%	95.7%
Positive predictive value	84.9%	87.7%	89.6%	91.3%
Negative predictive value	47.8%	42.2%	38.3%	33.3%

^a^Intra-amniotic inflammation was defined as elevated AF levels of IL-6 (≥1.5 ng/mL) and/or IL-8 (≥1.3 ng/mL).

^b^Sixteen women who were lost to follow-up were excluded from this analysis.

## Discussion

The principal findings of this study are as follows: (1) The frequency of short cervical length in women with pPROM was 29.2%; (2) short cervical length was independently associated with increased risk for intra-amniotic infection/inflammation; and (3) short cervical length was associated with an increased risk of impending preterm delivery, independent of the presence of intra-amniotic infection/inflammation. Specifically, in the current study, the specificities of a short cervix (≤15 mm) are 79.7% for intra-amniotic infection/inflammation and 83.3% for delivery within 7 days of measurement. These findings have important clinical implications, as cervical length assessment in pPROM, representing a less invasive test, can aid both physicians and patients in counseling to optimize fetal, maternal, and neonatal care, regarding decisions such as hospital admission, transfer to a tertiary center, amniocentesis, and administration of medications (i.e. corticosteroid, antibiotics, magnesium for neuroprotection).

An important finding of the current study is that a short cervical length is independently associated with an increased risk for intra-amniotic infection/inflammation in pPROM. This finding is consistent with the results of previous studies in the setting of preterm labor and intact membranes or an asymptomatic short cervix in the mid-trimester [11, 12, 22–24]. In fact, these observations are expected because a short cervical length may predispose to ascending infection from the vagina through the cervix into the uterus, which is known to be the most common pathway of intra-amniotic infection/inflammation, or vice versa [25]. In contrast to our results, Cobo et al. reported a lack of association between short cervical length and intra-amniotic infection/inflammation in a study investigating women with pPROM [26]. This discrepancy may be related to the difference in sample size (65 vs. 171); cervical length cutoffs used to define a short cervical length (≤25 mm vs. ≤15 mm); and gestational age at enrollment (<35 weeks vs. <34 weeks).

Of note, in the current study we found a significant association between a short cervix and intra-amniotic inflammation, but not intra-amniotic infection. However, this insignificant result regarding the relationship between a short cervix and intra-amniotic infection may be interpreted with caution (P = 0.081), as the power of this study was only 48% to detect a difference of 15.5% (a change from 32.5% to 48%) in the frequency of intra-amniotic infection. In addition, the utility of cervical length measurement as a single diagnostic test is limited due to its marginal association with intra-amniotic infection/inflammation (P = 0.034). Nevertheless, from a clinical perspective, the results of current study are important in that they highlight a less invasive technique to rapidly identify women at high risk for intra-amniotic infection/inflammation. Therefore, further studies are needed to determine whether and when sonographic cervical length might be clinically useful.

What is the plausible mechanism for the association of a short cervix with intra-amniotic infection/inflammation in pPROM? The most likely mechanisms for this association are as follows. First, intra-amniotic infection/inflammation may result from ascending infection of vaginal microbial flora across the cervix into the uterine cavity. Therefore, attenuation of the protective effect of the cervix against the vaginal pathogens can be exacerbated in the setting of cervical shortening in pPROM, in which the absence of fetal membranes along with a shortened cervix predispose to allow direct invasion of microorganisms into the amniotic cavity. Second, short cervical length may conversely produce pPROM as an end-point of the final pathway of preterm parturition, of which one of the definite causes is intra-amniotic infection/inflammation [27, 28].

Several studies have shown that short cervical length is associated with an increased risk of preterm delivery in pPROM [14–17, 29]. In accordance with previous studies, our results also showed that short cervical length increases the risks of preterm delivery within 2 days of measurement, 7 days of measurements, and before 34 weeks. Furthermore, multivariable analysis indicated that this association is independent of intra-amniotic infection/inflammation, which in both the current study and in the published literature is reported to be an important risk factor for impending preterm labor in pPROM [6, 16, 18, 26]. These findings underscore the importance of combining cervical length with inflammatory markers reflecting intra-amniotic infection/inflammation in the risk assessment for delivery latency following pPROM.

The findings that the overall incidences of a short cervix (≤15 mm) and delivery within 7 days of examination in our study population were 29.2% and 53.5%, respectively, are similar to the published data (29% and 48%, respectively) from another cohort of women with pPROM reported by Mehra et al [15]. The prevalence of intra-amniotic infection and intra-amniotic inflammation in our pPROM cohort were 36.8% and 54.4%, respectively; this is higher than those reported in previous studies by Shim et al. (23% and 42%, respectively) [6] and Cobo et al. (27.7% and 46.0%, respectively) [26] However, their assessment of AF for inflammatory status were conducted at a slightly later gestational age (<35 weeks of gestation) than the 21+0–33+6 weeks in our study, which may explain these differences given the reported inverse relationship between gestational age at assessment and the incidence of intra-amniotic infection/inflammation [6, 18, 26].

Our study has several limitations. First, the study was of retrospective nature and was based on data from a convenience sample of a tertiary care center, leading to a concern for lack of generalizability of the results. Second, for analysis of the current study, a cervical length of ≤15 mm was used to define short cervical length; the chosen cut-off for diagnosis of short cervical length may affect study results. Although the cut-off values to define a short cervical length were previously reported to be 15 to 25 mm [30], a cervical length of ≤15 mm in the general population has been proposed by several investigators as the threshold for treatment [31, 32]; for this reason, we used 15 mm to define short cervical length. Nevertheless, similar results were also obtained when data were analyzed at cervical length cutoffs of 10, 20, and 25 mm (Table 4). Third, the clinical value of AF IL-6 and IL-8 cut-offs to define intra-amniotic inflammation was not evaluated on the same patients in whom they were applied. Further studies are needed to confirm our findings in other populations in a prospective manner. Lastly, it is unclear from the current study how physicians can change clinical decisions based on the finding of a short cervix in the setting of pPROM although the result on a short cervix can inform physicians and patients about screening for intra-amniotic infection/inflammation and impending preterm delivery.

## Conclusions

In conclusion, in women with pPROM, a short cervical length is associated with an increased risk of intra-amniotic infection/inflammation and is strongly associated with impending preterm delivery, independent of the presence of intra-amniotic infection/inflammation. These findings support the importance of cervical length measurements in pPROM and the potential value of these measures as a less invasive means for identifying women at high-risk of pPROM-related adverse outcomes in which treatments might be targeted. Further prospective studies are needed to verify whether these associations are of a causal nature.

## Supporting information

S1 File Raw data. (SAV)
